# Intraoperative Use of Extracorporeal Membrane Oxygenation (ECMO) During Liver Transplantation: A Case Report

**DOI:** 10.7759/cureus.111553

**Published:** 2026-06-26

**Authors:** Tyler Lackland, Natalia Alston, Samantha Spence, Ziniya Shah, Caroline Farroll, Tyric Goode, Raymond I Okeke, Chintalapati R Varma, Henry B Randall, Govind Rangrass, Vidyaratna Fleetwood, Mustafa Nazzal

**Affiliations:** 1 Department of Surgery, Saint Louis University School of Medicine, St. Louis, USA; 2 Department of Acute Care Surgery, University of Missouri, Columbia, USA; 3 Department of Surgery, SSM Health Saint Louis University Hospital, St. Louis, USA; 4 Department of Anesthesiology and Critical Care, Saint Louis University School of Medicine, St. Louis, USA

**Keywords:** abdominal transplant surgery, artificial lung bypass, intraoperative ecmo, liver transplantation, veno-venous extracorporeal membrane oxygenation (vv ecmo)

## Abstract

Extracorporeal membrane oxygenation (ECMO) is increasingly used for cardiorespiratory support in hemodynamically unstable patients, yet standardized guidelines for its intraoperative use during liver transplantation are lacking. We present a retrospective review of two orthotopic liver transplant (OLT) recipients who required intraoperative veno-venous (VV) ECMO support.

The first patient, a 52-year-old female with alcohol-related cirrhosis, developed acute pulmonary edema, rising oxygen requirements, and oliguric kidney injury, necessitating emergent VV ECMO during OLT. The second patient, a 66-year-old male with nonalcoholic steatohepatitis-related cirrhosis and severe hepatopulmonary syndrome, underwent planned intraoperative VV ECMO to optimize oxygenation. Both patients tolerated ECMO well and recovered with stable postoperative liver function and respiratory status at follow-up.

These cases illustrate that intraoperative ECMO - whether emergent or planned - can be a valuable adjunct to manage severe cardiopulmonary instability during liver transplantation. Our findings support broader discussion and development of protocols guiding their use in this setting.

## Introduction

Extracorporeal membrane oxygenation (ECMO) is a modality used to support patients with cardiopulmonary instability [1]. Originally developed as a rescue therapy for severe respiratory or cardiac failure, its role has expanded to include optimization and bridging strategies for patients awaiting liver transplants [2]. ECMO is broadly categorized into veno-venous (VV) support for isolated pulmonary failure and veno-arterial (VA) support for combined cardiopulmonary failure [1, 3].

The use of ECMO in liver transplantation presents unique challenges. Patients with end-stage liver disease often have profound coagulopathy, portal hypertension, multiorgan dysfunction, and increased susceptibility to infection, all of which may be exacerbated by ECMO support and its associated anticoagulation requirements [3]. Consequently, perioperative ECMO has historically been considered high risk in this population. As such, VV ECMO has most commonly been described in the setting of post-transplant respiratory failure, often secondary to hepatopulmonary syndrome (HPS) [3]. However, severe respiratory failure before or during orthotopic liver transplantation (OLT) may preclude safe transplantation and is associated with substantial morbidity and mortality, creating a clinical scenario in which ECMO may represent the only viable means of maintaining transplant candidacy or facilitating successful graft implantation.

Despite its potential utility, perioperative VV ECMO remains rarely reported in liver transplantation, with no standardized guidelines and only sparse literature describing indications, timing, and outcomes. Existing evidence is largely limited to case reports and small retrospective series. In one of the largest published experiences, Patel et al. reported 29 patients who received perioperative VV ECMO, with 51% surviving to hospital discharge, suggesting that this approach may be feasible in select patients [4]. Nevertheless, the rarity of these cases and heterogeneity of reported indications have limited broader adoption and left many practical questions unanswered.

Here, we present two distinct cases of perioperative VV ECMO use during OLT at our institution. The first describes emergent VV ECMO initiation in a patient with acute-on-chronic liver failure, acute kidney injury (AKI) requiring continuous renal replacement therapy (CRRT), and severe pulmonary edema while awaiting transplantation. The second illustrates a separate clinical scenario requiring perioperative respiratory support to facilitate successful transplantation. Together, these cases highlight the potential role of VV ECMO as a bridge to and through liver transplantation in critically ill patients and add to the growing body of evidence supporting its use in carefully selected circumstances. Through this report, we aim to contribute practical experience that may inform future studies and help guide the management of similarly complex transplant candidates. 

## Case presentation

Case 1

Patient 1 was a 52-year-old female with a past medical history significant for alcoholic cirrhosis complicated by portal hypertension, hepatic encephalopathy, and ascites requiring large-volume paracentesis. She was diagnosed approximately one year before liver transplant after an episode of jaundice and abdominal distension, at which time she was referred for transplant evaluation. One month before the transplant, she had a Model for End-Stage Liver Disease with sodium (MELD-Na) [5] score of 30. Following evaluation in the emergency department for anemia, the patient was admitted to the hospital with hyperkalemia of 6.2 mmol/L (normal range: 3.5-5.2 mmol/L), before she was able to complete the necessary workup for transplant listing. Though the hyperkalemia soon resolved with conservative medical management, the patient experienced worsening hyponatremia accompanied by lethargy and encephalopathy, resulting in the need for transfer to the intensive care unit (ICU) for management of her deteriorating renal function.

While in the ICU, the patient developed an oliguric acute kidney injury, concerning for developing Type 1 hepatorenal syndrome. Continuous renal replacement therapy (CRRT) was initiated due to worsening AKI, indicated by worsening creatinine from 2.29 to 2.39 mg/dL, blood urea nitrogen (BUN) of 48 mg/dL, and worsening encephalopathy likely secondary to uremic complications. Volume status remained elevated despite a trial of midodrine and furosemide; nephrology recommended initiation of CRRT to assist with volume status and electrolyte balancing in the setting of decreased urine output. Acid-base status at the time of CRRT initiation was pH 7.42, arterial pressure of carbon dioxide (pCO_2_) was 39 mmHg, bicarbonate (HCO_3_) was 25.3 mmol/L, and arterial pressure of oxygen (paO_2_) was 100%. The patient was found to have increased pulmonary infiltrates in the setting of hypoxia, concerning for developing pulmonary edema versus pneumonia on computed tomography (CT) of her chest (Figure 1).

**Figure 1:**
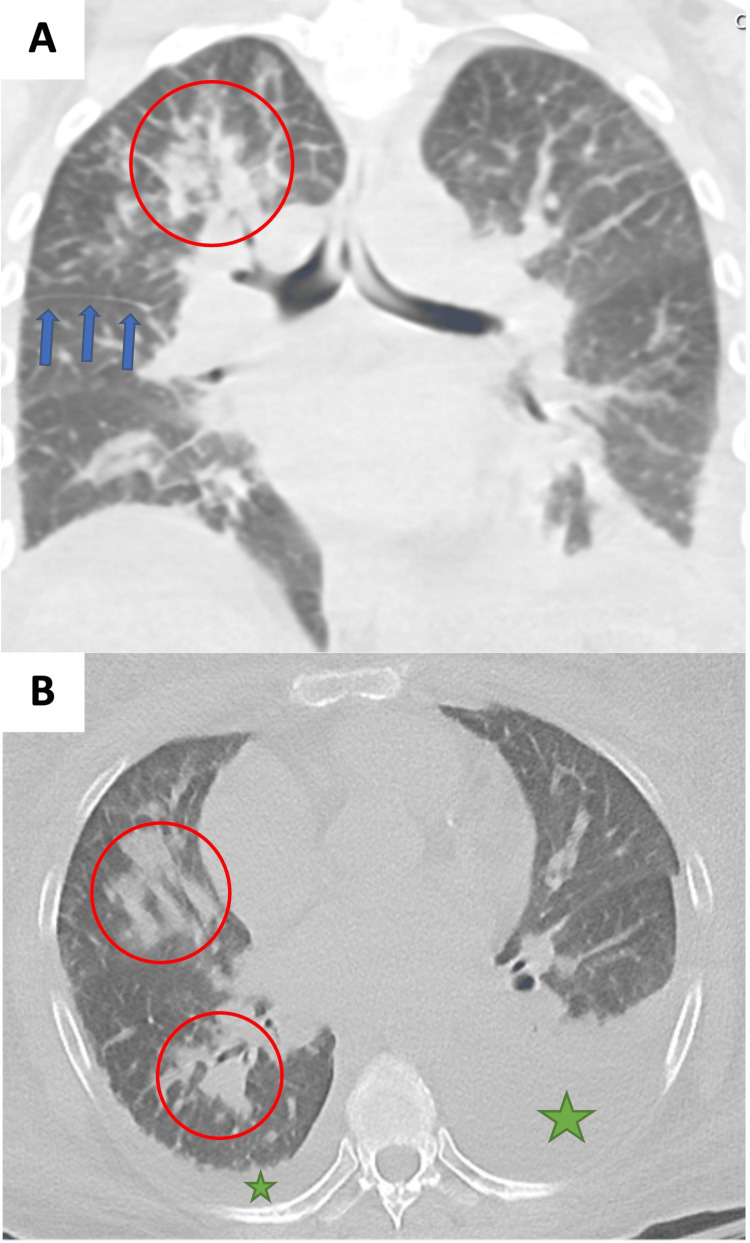
Computed Tomography (CT) With Contrast of Chest Patient 1 coronal (Panel A) and axial (Panel B) CT imaging of the chest acquired in the setting of persistent hypoxia, revealing interval progression of diffuse consolidations (red circles) with septal thickening (blue arrows) and worsening bilateral pleural effusions with atelectasis (green stars).

Pulmonary medicine was consulted for urgent bronchoscopy to rule out infection, as she was prepared to be placed on the liver transplant list that evening. She was mechanically ventilated on volume-controlled settings utilizing etomidate and rocuronium with a tidal volume of 450 mL and a respiratory rate of 15 breaths/minute. Despite relatively low ventilatory support requirements (positive end-expiratory pressure (PEEP) 5 cm H_2_O, minute ventilation 6.7 L/min), peak inspiratory pressures were elevated at 38 cm H_2_O, raising concern for increased airway resistance or impaired lung mechanics. She subsequently underwent a bronchoscopy with bronchoalveolar lavage (BAL), which cleared her airways of any retained secretions, and cultures were sent. Bronchoscopy with BAL demonstrated no endobronchial obstruction. BAL Gram stain and bacterial/fungal cultures were negative, with no organisms seen on stain and <10,000 CFU/mL of normal oropharyngeal flora, arguing against infectious pneumonia. She was successfully extubated following bronchoscopy to a 2L nasal cannula. Given the worsening clinical picture, the patient was urgently placed on the liver transplant list with a MELD-Na score of 41. The patient underwent a transplant three days after listing via the piggyback technique. 

Normothermic mechanical perfusion (NMP) of the donor's liver was utilized due to the anticipated long cold ischemia time (CIT) and concern for reperfusion injury in a critically ill patient with multiorgan failure. At the time of transplant, the patient was requiring vasopressor support in the ICU and was experiencing worsening oxygenation in the presence of pulmonary edema despite optimization of conventional ventilation. As such, the decision was made to place the patient on VV ECMO via the right internal jugular vein (IJV) (Figure 2) and the right iliac vein. Liver transplant proceeded, and the patient tolerated vascular exclusion, and the hepatectomy was completed. Intraoperative Doppler ultrasound demonstrated patent hepatic veins with appropriate flow direction and patent portal veins with normal hepatorenal flow. Patent hepatic arteries were visualized with brisk systolic upstroke and antegrade flow. There were no intraoperative complications, and the patient remained hemodynamically stable throughout the procedure on an appropriately decreasing infusion rate of norepinephrine. The estimated blood loss (EBL) was 500 mL, and the patient was transfused with 355 mL of cryoprecipitate, 600 mL of platelet pheresis, and 950 mL of leukoreduced red blood cells. CRRT was continued intraoperatively due to concern for fluid overload upon initiation of EMCO with subsequent worsening of pulmonary status [6]. The total intraoperative ultrafiltrate was 1700 mL. VV ECMO was weaned and successfully decannulated before the procedure ended. 

**Figure 2:**
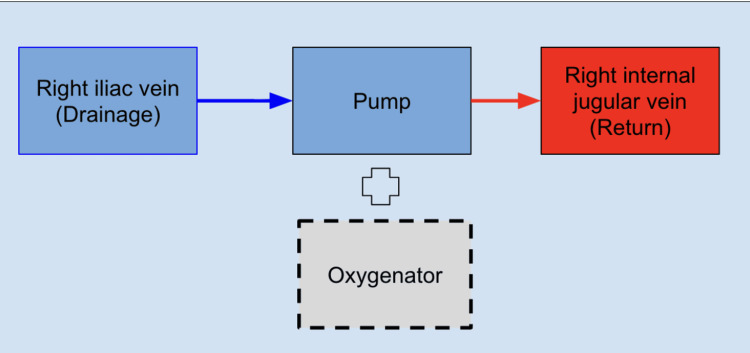
Modified VV ECMO With Added Oxygenation During the Anhepatic Phase of Liver Transplantation Modified veno-venous extracorporeal membrane oxygenation (VV ECMO) established via liver transplant veno-venous bypass circuit. Deoxygenated blood drains from the right iliac vein, passes through the centrifugal pump and attached oxygenator, and returns via the right internal jugular vein into the superior vena cava/right atrium. Red: oxygenated blood. Blue: deoxygenated blood. Image credit: Lackland T via BioRender

The patient’s lactate downtrended appropriately during the postoperative period from 2.4 to 1.1 mmol/L in the first 24 hours. Post-operative liver ultrasounds demonstrated normal sonographic appearance of the liver with elevated resistive indices throughout the hepatic and minimal diastolic flow likely secondary to postoperative edema (Figure 3). By postoperative day (POD) 3, the hepatic vasculature remained patent with improved resistance indices, signifying improved postoperative edema. On POD 5, the patient was successfully extubated, and CRRT was de-escalated to intermittent hemodialysis, which was ultimately discontinued at discharge on POD 15. Following discharge to home with home health, her labs continued to improve with alanine aminotransferase (ALT) decreasing from 58 U/L postoperatively to 12 U/L (normal range: 6-29 U/L) and aspartate aminotransferase (AST) remaining within normal limits with values ranging from 33-13 U/L (normal range: 10-35 U/L). Liver function testing indicated stable liver function postoperatively, which was reaffirmed on the most recent follow-up, seven months post-transplant.

**Figure 3:**
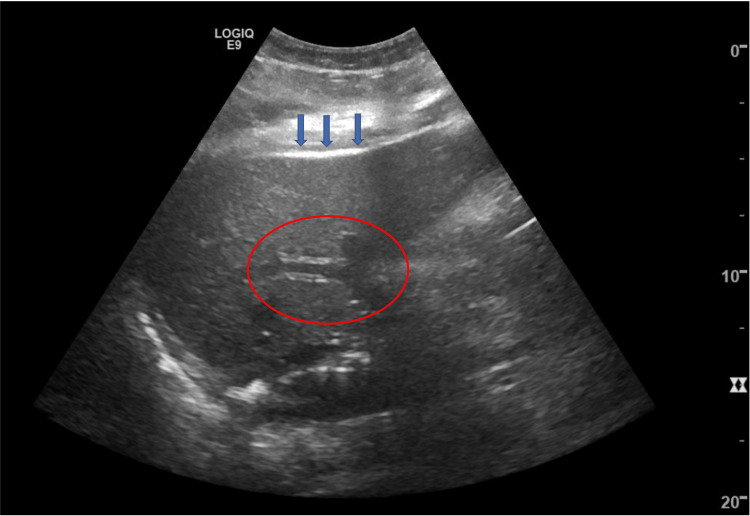
Patient 1 Postoperative Liver Ultrasound Liver ultrasound acquired post-transplantation demonstrating normal sonographic appearance and patent hepatic vasculature (red circle) with minimal perihepatic free fluid (blue arrows).

Case 2

Patient 2 was a 66-year-old male with a past medical history of end-stage liver disease secondary to nonalcoholic steatohepatitis complicated by hepatic encephalopathy, hepatocellular carcinoma status post thermal ablation one month prior, esophageal varices on propranolol, and severe hepatopulmonary syndrome (HPS) (defined as shunt fraction ≥20% with PaO_2_ < 60 mmHg [7]) At baseline, the patient required 15 L/min via nonrebreather due to chronic hypoxemia (PaO_2_ 45 mmHg on room air (normal range: 75-100 mmHg)). Pre-transplant transesophageal echocardiogram with agitated saline demonstrated an aneurysmal interatrial septum and predominant right-to-left extracardiac shunt, likely contributing to refractory hypoxemia. A detailed report can be found in our previous publication [8]. Arterial blood gas prior to transplant was persistently significant for alkalemia (arterial pH 7.46-7.49 (normal range: 7.35-7.45)) and hypocarbia (arterial carbon dioxide pressure (PaCO_2_) 23-26 mmHg (normal range: 35-45 mmHg)), consistent with chronic respiratory alkalosis. The patient had been on the transplant list for approximately 16 months before an organ became available via donation after brain death. At the time of listing, he had a MELD-Na score of 15 and a MELD-Na score of 13 upon admission. The patient was also noted to be hypotensive and was started on oral midodrine preoperatively. 

During his pre-transplant admission, the patient experienced progressively worsening hypoxemia due to severe HPS with significant right-to-left shunting, consistently requiring a minimum of 10L supplemental oxygen via a non-rebreather mask to maintain oxygen saturations above 90%. Given the degree of pre-transplant hypoxemia despite optimization of oxygen therapy, it was determined that there was a high likelihood that the patient would require mechanical support following surgery and likely supplemental oxygen for at least one year postoperatively. Ultimately, the decision was made to place the patient on VV ECMO preoperatively for oxygenation and circulation support in the setting of expected intraoperative physiologic stress, and the donor liver was placed on NMP due to the anticipated prolonged CIT. A 31/18 ProtekDuo bypass cannula (LivaNova PLC, London, UK) was placed into the right IJV (Figure 4) before the procedure started, and it remained in place throughout the operation. The patient underwent OLT using a bicaval technique.

**Figure 4:**
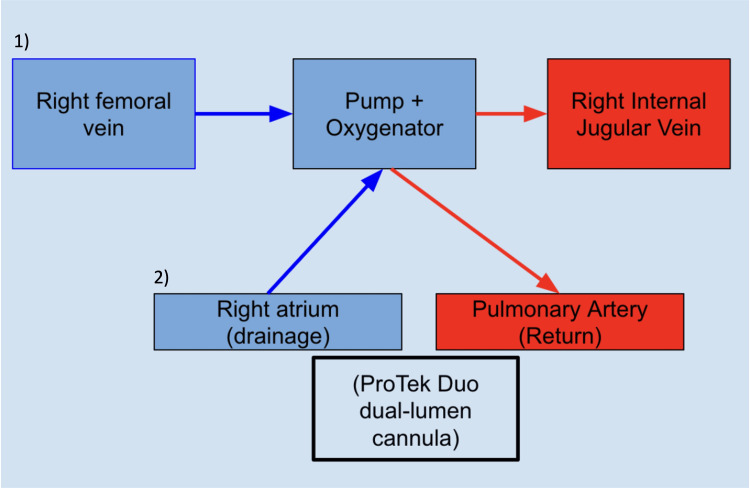
Veno-Venous ECMO Configuration via Right Femoral Vein and Right Internal Jugular Vein Schematic of veno-venous extracorporeal membrane oxygenation (ECMO) using a 1) right femoral cannula and 2) right internal jugular vein ProTek Duo dual-lumen cannula (LivaNova PLC, London, UK). 1) Deoxygenated blood drains from the right femoral vein, passes through the centrifugal pump and attached oxygenator, and returns via the right internal jugular vein into the superior vena cava/right atrium. 2) Deoxygenated blood drains from the right atrium through the proximal inflow port of the dual-lumen cannula, passes through the pump and oxygenator, and returns to the main pulmonary artery via the distal outflow port of the cannula. Red: oxygenated blood. Blue: deoxygenated blood. Image credit: Lackland T via BioRender

A second VV bypass cannula was placed in the right femoral vein (Figure 4) to maintain hemodynamic stability during inferior vena cava (IVC) clamping by preventing surrounding vessel collapse and general negative pressure within the IVC to facilitate hepatic drainage [3]. The VV bypass was decannulated after the portal vein, and the hepatic artery was anastomosed. Static CIT (time from cross-clamp to machine perfusion) was approximately 2 hours, and machine perfusion time was 8 hours and 40 minutes for a total preservation time of 10 hours and 41 minutes. The EBL was 3000 mL, and the patient was transfused with eight units of packed red blood cells and nine units of fresh frozen plasma intraoperatively. As the transplant concluded, the patient was transferred back to the ICU in stable condition, intubated, sedated, and on VV ECMO via the right IJV to maintain oxygenation. The patient continued to require pressors, norepinephrine, and vasopressin postoperatively due to persistent hypotension and was weaned as tolerated.

The patient’s hepatic ultrasound performed on POD 1 revealed patent hepatic vasculature without evidence of perihepatic hematoma or fluid collection (Figure 5). However, the patient’s postoperative course was complicated by a persistently high sanguineous output from his abdominal drains and associated anemia unresponsive to blood transfusion, concerning for postoperative bleeding. Thromboelastography (TEG) was performed and demonstrated inhibition of coagulation consistent with reduced clotting efficiency. Though likely multifactorial, it is possible that the systemic coagulation required to maintain ECMO circuit patency may have contributed to impaired hemostasis and increased bleeding risk. However, as the bleeding persisted despite coagulopathy correction with TEG-guided resuscitation, the bleeding issue likely superseded that of anticoagulation and warranted surgical evaluation. The patient was subsequently taken back to the operating room on POD 3 for exploratory laparotomy and abdominal washout, during which significant arterial bleeding from a lymph node adjacent to the bile duct was identified and corrected.

**Figure 5:**
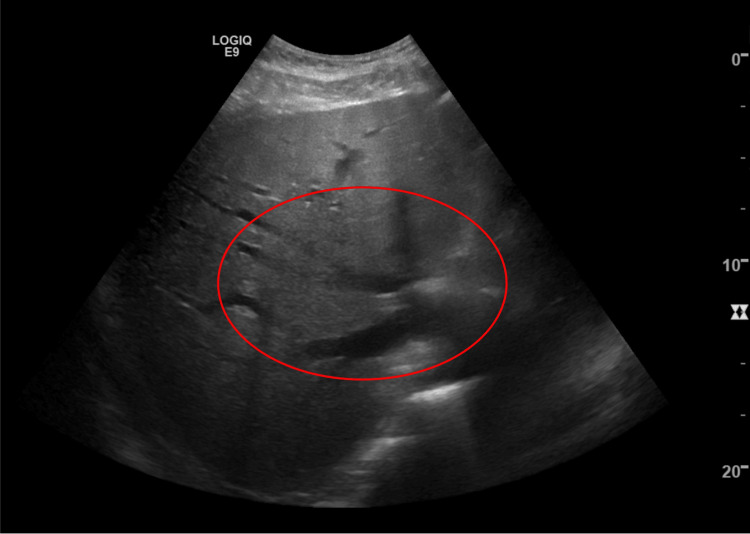
Case 2 Postoperative Liver Ultrasound Liver ultrasound acquired post-transplantation demonstrating normal sonographic appearance and patent hepatic vasculature (red circle) without perihepatic hematoma or fluid collection.

On POD 6, the patient was noted to have abnormal liver function tests in a cholestatic pattern, and a hepatobiliary iminodiacetic acid (HIDA) scan revealed no radiotracer activity within the biliary system or bowel, concerning for common bile duct (CBD) obstruction (Figure 6). Endoscopic retrograde cholangiopancreatography (ERCP) confirmed the presence of a mild anastomotic stricture, and a biliary stent was placed in the CBD. The patient was also experiencing prolonged ventilatory requirements, requiring tracheostomy. VV ECMO was decannulated on POD 11, and the patient was tolerating an intermittent tracheostomy collar prior to discharge to a long-term acute care hospital 28 days post-transplantation. The patient’s tracheostomy was subsequently decannulated 3 weeks after discharge. At his most recent follow-up appointment, twenty months post-transplant, the patient was maintaining good respiratory status, and his labs exhibited excellent graft function.

**Figure 6:**
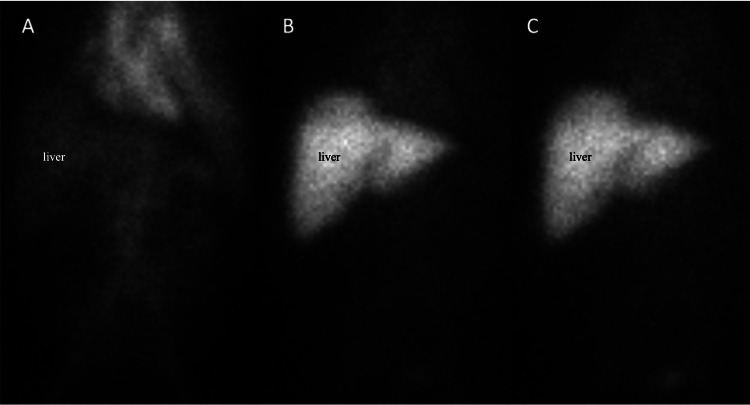
Case 2 Postoperative HIDA Scan Hepatobiliary iminodiacetic acid (HIDA) scan acquired post-transplantation in the setting of abnormal liver function tests demonstrating delayed radiotracer (white) clearance from the liver with the absence of radiotracer activity within the biliary system or bowel at minute 0 (Panel A), minute 30 (Panel B), and minute 60 (Panel C).

## Discussion

Veno-venous and veno-arterial ECMO in the liver transplant patient can serve as a supportive therapy postoperatively as well as a bridge to transplant therapy preoperatively. When cardiac and respiratory support is needed, utilization of veno-arterial ECMO is preferred. Conversely, when only respiratory support is required, veno-venous ECMO can be utilized [2]. ECMO was first used in the early 2000s for liver transplant patients as a rescue therapy for severe cardiopulmonary collapse following transplant [9]. Recent literature has assessed its utilization in the preoperative period as a bridging therapy to transplant in individuals with reversible cardiopulmonary compromise [10]. Prior case reports discussed the use of preoperative VA and VV ECMO as a bridge therapy for patients with severe to moderate portopulmonary hypertension, heart failure, acute respiratory distress syndrome, and refractory hypoxemia. With mortality as the primary outcome, the majority of individuals in each case report were alive at the time of publication and survived to 20-month postoperative follow-up [2]. 

In both of our patients' cases, ECMO was utilized intraoperatively to avoid intraoperative and early postoperative hypoxemia in the setting of fluid overload concerns in the presence of acute kidney injury requiring CRRT and severe HPS, respectively. In our first case, VV ECMO was initiated emergently in the operating room, and in the second case, it was planned due to severe HPS. Initiation of ECMO required extensive risk/benefit analysis, given the many risks associated with ECMO. A prior study found that implementation of ECMO during liver transplant improved mortality by 40% in patients who were otherwise not expected to survive [3]. This same study proposed that candidates for ECMO should have reversible causes of severe, acute cardiac and/or respiratory failure that are refractory to conventional ventilatory and resuscitative efforts. For patients with reversible pulmonary and cardiopulmonary pathologies, ECMO was found to improve the survival rate to 82%, specifically in those with HPS [2]. In general, VV ECMO can be used for respiratory failure in the setting of OLT due to intrapulmonary shunting in HPS, pneumonia, or acute respiratory distress syndrome during the perioperative period. On the other hand, VA ECMO can be used for reversible cardiovascular collapse secondary to massive pulmonary embolism, intracardiac thrombus, right heart failure, air embolus, or septic shock [3]. There are also new findings suggesting that earlier implementation of ECMO for liver transplant patients may invoke better outcomes, compared to the more widely known utilization as a salvage therapy [2,11]. 

Currently, ECMO utilization in the perioperative period lacks a true protocol and is on a risk/benefit basis. While there is extensive research on the postoperative use of ECMO in liver transplant patients, understanding of its use as a bridging therapy continues to grow. Current literature has identified that the candidates most suitable for ECMO are those with reversible, acute cardiopulmonary collapse, as seen in both of our patients. Following successful decannulation, both of our patients have done well postoperatively. Our 66-year-old male patient, despite a relatively complicated hospital course, has had excellent graft function and was most recently seen 16 months post-op with improved baseline pulmonary status. Our 52-year-old female patient presented well at her 6-month follow-up with both improved renal function and lab values. 

This study is limited by its retrospective case report design, inclusion of only two patients from a single institution, and lack of a control group, all of which limit generalizability and preclude assessment of the independent effect of VV ECMO on outcomes. The heterogeneity of the cases further restricts conclusions regarding optimal patient selection, timing of initiation, cannulation strategies, or duration of support. VV ECMO in the liver transplant setting also carries important risks, including severe bleeding related to systemic anticoagulation in the context of underlying coagulopathy, as well as potential vascular injury, circuit thrombosis, and increased infection risk. Accordingly, the decision to initiate ECMO should be individualized, balancing risks and benefits based on patient-specific clinical factors and expert clinical judgement. Nevertheless, our findings suggest that, in carefully selected cases, perioperative VV ECMO may represent a promising adjunct in liver transplantation, warranting further evaluation in larger multicenter studies.

## Conclusions

It is our goal with this case report to add to the growing body of literature surrounding ECMO use as a bridging therapy for OLT patients. ECMO is currently utilized on a case-by-case basis depending on patient status. Though planned ECMO has been previously described in the perioperative period in severe cases of hepato-pulmonary syndrome, our experience shows a possible role for emergent ECMO in patients with acute-on-chronic liver failure and acute kidney injury requiring CRRT in the presence of pulmonary edema and the need for urgent liver transplantation. 

As literature continues to grow, it will be important to conduct retrospective and prospective analyses to further investigate its use, risk/benefit, long-term outcomes, and overall benefits in post-transplant outcomes.
